# Supplementary material for: EvatCrop: a novel hybrid quasi-fuzzy artificial neural network (ANN) model for estimation of reference evapotranspiration
Source: PeerJ. 2024 May 31;12:e17437. doi: 10.7717/peerj.17437 (PMC11146332; doi:10.7717/peerj.17437)
Supplement: Supplemental Information 2 [file peerj-12-17437-s002.docx]

**Table 1.** Statistical measures for meteorological parameters of the dataset.

| Location |  |  | *T_min_* | *T_max_* | *W_s_* | *R_h_* | *S_r_* | *ET*_0_ |
| --- | --- | --- | --- | --- | --- | --- | --- | --- |
|  |  |  | (°C) | (°C) | (m/s) | % | (*MJm*^−^2 *d*^−^1) | (mm/d) |
| Berubari | Training set | Max | 30.71 | 47.49 | 6.05 | 0.97 | 29.34 | 12.55 |
|  |  | Min | 3.45 | 15.52 | 0.58 | 0.07 | 0.41 | 1.822 |
|  |  | Sd | 6.43 | 4.78 | 0.67 | 0.22 | 5.67 | 2.07 |
|  |  | Cv | 0.31 | 0.14 | 0.37 | 0.36 | 0.30 | 0.35 |
|  |  | Sk | -0.70 | 0.19 | 0.92 | -0.39 | -0.85 | 0.33 |
|  | Testing set | Max | 28.50 | 47.37 | 4.37 | 0.97 | 28.56 | 10.98 |
|  |  | Min | 2.91 | 15.44 | 0.60 | 0.09 | 0.29 | 2.00 |
|  |  | Sd | 6.03 | 4.83 | 0.61 | 0.25 | 6.90 | 1.78 |
|  |  | Cv | 0.30 | 0.15 | 0.32 | 0.41 | 0.39 | 0.30 |
|  |  | Sk | -0.67 | 0.22 | 0.86 | -0.39 | -0.76 | 0.35 |
| Jayanti | Training set | Max | 24.58 | 37.09 | 5.00 | 0.98 | 29.64 | 10.48 |
|  |  | Min | 0.81 | 12.13 | 0.62 | 0.08 | 0.51 | 1.83 |
|  |  | Sd | 5.64 | 3.89 | 0.54 | 0.22 | 6.37 | 1.35 |
|  |  | Cv | 0.35 | 0.14 | 0.30 | 0.33 | 0.35 | 0.26 |
|  |  | Sk | -0.44 | -0.18 | 0.48 | -0.58 | -0.67 | 0.44 |
|  | Testing set | Max | 25.32 | 39.53 | 3.50 | 0.98 | 29.20 | 9.59 |
|  |  | Min | 2.40 | 14.32 | 0.54 | 0.08 | 0.59 | 2.00 |
|  |  | Sd | 5.09 | 3.89 | 0.52 | 0.25 | 6.72 | 1.32 |
|  |  | Cv | 0.29 | 0.14 | 0.30 | 0.41 | 0.37 | 0.25 |
|  |  | Sk | -0.69 | -0.19 | 0.09 | -0.36 | -0.62 | 0.19 |
| Tamaguri | Training set | Max | 29.24 | 45.18 | 6.42 | 0.98 | 29.13 | 11.13 |
|  |  | Min | 1.20 | 13.84 | 0.52 | 0.12 | 0.41 | 1.79 |
|  |  | Sd | 5.79 | 4.46 | 0.55 | 0.21 | 5.65 | 1.57 |
|  |  | Cv | 0.29 | 0.14 | 0.35 | 0.32 | 0.31 | 0.30 |
|  |  | Sk | -0.62 | 0.16 | 1.54 | -0.51 | -0.85 | 0.35 |
|  | Testing set | Max | 27.64 | 44.83 | 4.49 | 0.98 | 28.32 | 9.44 |
|  |  | Min | 2.92 | 17.97 | 0.58 | 0.12 | 0.62 | 2.13 |
|  |  | Sd | 5.47 | 4.40 | 0.55 | 0.23 | 6.43 | 1.52 |
|  |  | Cv | 0.27 | 0.14 | 0.34 | 0.34 | 0.38 | 0.29 |
|  |  | Sk | -0.66 | 0.35 | 1.25 | -0.64 | -0.66 | 0.23 |
