# Supplementary material for: EvatCrop: a novel hybrid quasi-fuzzy artificial neural network (ANN) model for estimation of reference evapotranspiration
Source: PeerJ. 2024 May 31;12:e17437. doi: 10.7717/peerj.17437 (PMC11146332; doi:10.7717/peerj.17437)
Supplement: Supplemental Information 3 [file peerj-12-17437-s003.docx]

**Table 2.** Eight input combinations of five meteorological parameters.

| Sl. No. | Input combinations | Meteorological parameters |
| --- | --- | --- |
| 1. | *C1* | *T_min_*, *T_max_* |
| 2. | *C2* | *T_min_* , *T_max_*, *W_s_* |
| 3. | *C3* | *T_min_*, *T_max_*, *R_h_* |
| 4. | *C4* | *T_min_*, *T_max_*, *S_r_* |
| 5. | *C5* | *T_min_*, *T_max_*, *W_s_*, *R_h_* |
| 6. | *C6* | *T_min_*, *T_max_*, *W_s_*, *S_r_* |
| 7. | *C7* | *T_min_*, *T_max_*, *R_h_*, *S_r_* |
| 8. | *C8* | *T_min_*, *T_max_*, *W_s_*, *R_h_*, *S_r_* |
