# Supplementary material for: EvatCrop: a novel hybrid quasi-fuzzy artificial neural network (ANN) model for estimation of reference evapotranspiration
Source: PeerJ. 2024 May 31;12:e17437. doi: 10.7717/peerj.17437 (PMC11146332; doi:10.7717/peerj.17437)
Supplement: Supplemental Information 4 [file peerj-12-17437-s004.docx]

**Table 3.** Input combinations, rule groups, number of rules and the interim output vectors.

| Input Combinations | Rule groups | Number of rules | Interim output vectors |
| --- | --- | --- | --- |
| *C*1={*T_min_*, *T_max_*} | *RG*1 | 8 | {*I*}_1_ |
| *C*2={*T_min_*, *T_max_*, *W_s_*} | *RG*2 | 27 | {*I*}_2_ |
| *C*3={*T_min_*, *T_max_*, *R_h_*} | *RG*3 | 27 | {*I*}_3_ |
| *C*4={*T_min_* ,*T_max_*, *S_r_*} | *RG*4 | 27 | {*I*}_4_ |
| *C*5={*T_min_*, *T_max_*, *W_s_*, *R_h_*} | *RG*5 | 81 | {*I*}_5_ |
| *C*6={*T_min_*, *T_max_*, *W_s_*, *S_r_*} | *RG*6 | 81 | {*I*}_6_ |
| *C*7={*T_min_*, *T_max_*, *R_h_*, *S_r_*} | *RG*7 | 81 | {*I*}_7_ |
| *C*8={*T_min_*, *T_max_*, *W_s_*, *R_h_*, S*_r_*} | *RG*8 | 243 | {*I*}_8_ |
