# Supplementary material for: EvatCrop: a novel hybrid quasi-fuzzy artificial neural network (ANN) model for estimation of reference evapotranspiration
Source: PeerJ. 2024 May 31;12:e17437. doi: 10.7717/peerj.17437 (PMC11146332; doi:10.7717/peerj.17437)
Supplement: Supplemental Information 5 [file peerj-12-17437-s005.docx]

**Table 4.** Optimum number of hidden layer neurons and activation functions of eight MLPs used against eight input combinations for three study locations.

| Locations | Input combinations | MLPs | Number of nodes in input layer | Number of nodes in hidden layer | Activation functions |
| --- | --- | --- | --- | --- | --- |
| Berubari | *C*1  *C*2  *C*3  *C*4  *C*5  *C*6  *C*7  *C*8 | MLP_1_  MLP_2_  MLP_3_  MLP_4_  MLP_5_  MLP_6_  MLP_7_  MLP_8_ | 8  27  27  27  81  81  81  243 | 5  7  8  12  11  6  15  9 | Sigmoid  Tangent hyperbolic  Tangent hyperbolic  Tangent hyperbolic  Tangent hyperbolic  Tangent hyperbolic  Tangent hyperbolic  Tangent hyperbolic |
| Jayanti | *C*1  *C*2  *C*3  *C*4  *C*5  *C*6  *C*7  *C*8 | MLP_1_  MLP_2_  MLP_3_  MLP_4_  MLP_5_  MLP_6_  MLP_7_  MLP_8_ | 8  27  27  27  81  81  81  243 | 10  11  10  6  7  7  6  6 | Tangent hyperbolic  Tangent hyperbolic  Tangent hyperbolic  Sigmoid  Tangent hyperbolic  Tangent hyperbolic  Tangent hyperbolic  Tangent hyperbolic |
| Tamaguri | *C*1  *C*2  *C*3  *C*4  *C*5  *C*6  *C*7  *C*8 | MLP_1_  MLP_2_  MLP_3_  MLP_4_  MLP_5_  MLP_6_  MLP_7_  MLP_8_ | 8  27  27  27  81  81  81  243 | 14  10  7  14  8  10  6  8 | Tangent hyperbolic  Tangent hyperbolic  Tangent hyperbolic  Sigmoid  Tangent hyperbolic  Sigmoid  Tangent hyperbolic  Tangent hyperbolic |
