# Supplementary material for: EvatCrop: a novel hybrid quasi-fuzzy artificial neural network (ANN) model for estimation of reference evapotranspiration
Source: PeerJ. 2024 May 31;12:e17437. doi: 10.7717/peerj.17437 (PMC11146332; doi:10.7717/peerj.17437)
Supplement: Supplemental Information 6 [file peerj-12-17437-s006.docx]

**Table 5.** The experimental values of the performance metrics obtained for the training set of Berubari.

| **Input**  **combinations** | **Models** | *R*2 | *d* | *Ag* | *RMSE* | *RMSRE* | *Ae* |
| --- | --- | --- | --- | --- | --- | --- | --- |
|  | DT | 0.636 | 0.879 | 0.757 | 1.247 | 0.240 | 0.744 |
| *C1* | ANN  ANFIS | 0.619  0.623 | 0.870  0.873 | 0.744  0.748 | 1.277  1.270 | 0.250  0.240 | 0.763  0.755 |
|  | *EvatCrop* | **0.643** | **0.882** | **0.762** | **1.236** | **0.236** | **0.736** |
|  | DT | 0.982 | 0.996 | 0.989 | 0.274 | 0.054 | 0.164 |
| *C2* | ANN  ANFIS | 0.972  0.984 | 0.993  0.996 | 0.983  0.990 | 0.343  0.264 | 0.071  0.051 | 0.207  0.157 |
|  | *EvatCrop* | **0.985** | **0.996** | **0.990** | **0.256** | **0.049** | **0.153** |
|  | DT | 0.743 | 0.922 | 0.833 | 1.047 | 0.194 | 0.621 |
| *C3* | ANN  ANFIS | 0.710  0.747 | 0.909  0.923 | 0.809  0.835 | 1.114  1.040 | 0.202  0.188 | 0.658  0.614 |
|  | *EvatCrop* | **0.756** | **0.927** | **0.841** | **1.021** | **0.185** | **0.603** |
|  | DT | 0.703 | 0.907 | 0.805 | 1.127 | 0.212 | 0.669 |
| *C4* | ANN  ANFIS | 0.658  0.705 | 0.887  0.907 | 0.773  0.806 | 1.209  1.124 | 0.232  0.209 | 0.720  0.666 |
|  | *EvatCrop* | **0.721** | **0.914** | **0.817** | **1.092** | **0.201** | **0.647** |
|  | DT | 0.983 | 0.996 | 0.989 | 0.272 | 0.054 | 0.163 |
| *C5* | ANN  ANFIS | 0.977  0.987 | 0.994  0.997 | 0.986  0.992 | 0.312  0.232 | 0.061  0.045 | 0.187  0.138 |
|  | *EvatCrop* | **0.988** | **0.997** | **0.992** | **0.231** | **0.044** | **0.137** |
|  | DT | 0.995 | 0.999 | 0.997 | 0.146 | 0.028 | 0.087 |
| *C6* | ANN  ANFIS | 0.983  0.994 | 0.996  0.999 | 0.989  0.996 | 0.269  0.157 | 0.060  0.029 | 0.165  0.093 |
|  | *EvatCrop* | **0.995** | **0.999** | **0.997** | **0.140** | **0.025** | **0.082** |
|  | DT | 0.768 | 0.931 | 0.849 | 0.996 | 0.183 | 0.590 |
| *C7* | ANN  ANFIS | 0.730  0.817 | 0.918  0.948 | 0.824  0.882 | 1.075  0.884 | 0.204  0.156 | 0.639  0.520 |
|  | *EvatCrop* | **0.821** | **0.949** | **0.885** | **0.874** | **0.155** | **0.515** |
|  | DT | 0.995 | 0.999 | 0.997 | 0.143 | 0.028 | 0.086 |
| *C8* | ANN  ANFIS | 0.968  0.996 | 0.992  0.999 | 0.980  0.998 | 0.371  0.127 | 0.090  0.024 | 0.230  0.075 |
|  | *EvatCrop* | **0.996** | **0.999** | **0.998** | **0.123** | **0.022** | **0.072** |

**RMSE* measured in mm/day.
