# Supplementary material for: EvatCrop: a novel hybrid quasi-fuzzy artificial neural network (ANN) model for estimation of reference evapotranspiration
Source: PeerJ. 2024 May 31;12:e17437. doi: 10.7717/peerj.17437 (PMC11146332; doi:10.7717/peerj.17437)
Supplement: Supplemental Information 7 [file peerj-12-17437-s007.docx]

**Table 6.** The experimental values of the performance metrics obtained for the testing set of Berubari.

| **Input**  **combinations** | **Models** | *R*2 | *d* | *Ag* | *RMSE* | *RMSRE* | *Ae* |
| --- | --- | --- | --- | --- | --- | --- | --- |
|  | DT | 0.473 | 0.826 | 0.650 | 1.305 | 0.224 | 0.764 |
| *C*1 | ANN  ANFIS | 0.502  0.491 | 0.830  0.831 | 0.666  0.661 | 1.269  1.282 | 0.218  0.222 | 0.743  0.752 |
|  | *EvatCrop* | **0.511** | **0.840** | **0.675** | **1.257** | **0.216** | **0.737** |
|  | DT | 0.954 | 0.988 | 0.971 | 0.385 | 0.071 | 0.228 |
| *C*2 | ANN  ANFIS | 0.958  0.955 | 0.989  0.988 | 0.974  0.972 | 0.369  0.380 | 0.068  0.071 | 0.218  0.225 |
|  | *EvatCrop* | **0.970** | **0.992** | **0.981** | **0.313** | **0.054** | **0.184** |
|  | DT | 0.501 | 0.843 | 0.672 | 1.269 | 0.205 | 0.737 |
| *C*3 | ANN  ANFIS | 0.558  0.516 | 0.853  0.844 | 0.705  0.680 | 1.195  1.251 | 0.197  0.212 | 0.696  0.731 |
|  | *EvatCrop* | **0.578** | **0.863** | **0.720** | **1.168** | **0.193** | **0.681** |
|  | DT | 0.562 | 0.867 | 0.714 | 1.189 | 0.213 | 0.701 |
| *C*4 | ANN  ANFIS | 0.561  0.591 | 0.859  0.875 | 0.710  0.733 | 1.191  1.150 | 0.219  0.208 | 0.705  0.679 |
|  | *EvatCrop* | **0.619** | **0.883** | **0.751** | **1.109** | **0.200** | **0.654** |
|  | DT | 0.947 | 0.986 | 0.967 | 0.412 | 0.073 | 0.242 |
| *C*5 | ANN  ANFIS | 0.963  0.959 | 0.991  0.990 | 0.977  0.974 | 0.344  0.362 | 0.060  0.067 | 0.202  0.215 |
|  | *EvatCrop* | **0.973** | **0.993** | **0.983** | **0.294** | **0.053** | **0.173** |
|  | DT | 0.964 | 0.991 | 0.977 | 0.343 | 0.060 | 0.201 |
| *C*6 | ANN  ANFIS | 0.975  0.980 | 0.993  0.995 | 0.984  0.987 | 0.287  0.256 | 0.059  0.047 | 0.173  0.151 |
|  | *EvatCrop* | **0.988** | **0.997** | **0.992** | **0.205** | **0.035** | **0.120** |
|  | DT | 0.584 | 0.877 | 0.731 | 1.159 | 0.203 | 0.681 |
| *C*7 | ANN  ANFIS | 0.645  0.436 | 0.887  0.843 | 0.766  0.639 | 1.071  1.350 | 0.187  0.279 | 0.629  0.814 |
|  | *EvatCrop* | **0.683** | **0.902** | **0.793** | **1.012** | **0.173** | **0.592** |
|  | DT | 0.962 | 0.990 | 0.976 | 0.348 | 0.062 | 0.205 |
| *C*8 | ANN  ANFIS | 0.957  0.440 | 0.989  0.875 | 0.973  0.658 | 0.371  1.344 | 0.079  0.298 | 0.225  0.821 |
|  | *EvatCrop* | **0.988** | **0.997** | **0.992** | **0.198** | **0.034** | **0.116** |

**RMSE* measured in mm/day.
