# Supplementary material for: EvatCrop: a novel hybrid quasi-fuzzy artificial neural network (ANN) model for estimation of reference evapotranspiration
Source: PeerJ. 2024 May 31;12:e17437. doi: 10.7717/peerj.17437 (PMC11146332; doi:10.7717/peerj.17437)
Supplement: Supplemental Information 8 [file peerj-12-17437-s008.docx]

**Table 7.** The experimental values of the performance metrics obtained for the training set of Jayanti.

| **Input**  **combinations** | **Models** | *R*2 | *d* | *Ag* | *RMSE* | *RMSRE* | *Ae* |
| --- | --- | --- | --- | --- | --- | --- | --- |
|  | DT | 0.651 | 0.886 | 0.769 | 0.798 | 0.169 | 0.484 |
| *C1* | ANN  ANFIS | 0.616  0.664 | 0.870  0.891 | 0.743  0.777 | 0.838  0.784 | 0.181  0.166 | 0.509  0.475 |
|  | *EvatCrop* | **0.668** | **0.893** | **0.781** | **0.779** | **0.164** | **0.471** |
|  | DT | **0.956** | **0.989** | **0.972** | **0.284** | **0.057** | **0.171** |
| *C2* | ANN  ANFIS | 0.935  0.950 | 0.983  0.987 | 0.959  0.969 | 0.344  0.302 | 0.069  0.058 | 0.207  0.180 |
|  | *EvatCrop* | 0.953 | 0.988 | 0.970 | 0.294 | 0.057 | 0.176 |
|  | DT | 0.782 | 0.936 | 0.859 | 0.631 | 0.131 | 0.381 |
| *C3* | ANN  ANFIS | 0.734  0.804 | 0.919  0.943 | 0.826  0.874 | 0.697  0.598 | 0.142  0.123 | 0.419  0.360 |
|  | *EvatCrop* | **0.814** | **0.947** | **0.880** | **0.583** | **0.120** | **0.351** |
|  | DT | 0.747 | 0.923 | 0.835 | 0.679 | 0.140 | 0.410 |
| *C4* | ANN  ANFIS | 0.732  0.763 | 0.919  0.929 | 0.825  0.846 | 0.699  0.658 | 0.144  0.134 | 0.421  0.396 |
|  | *EvatCrop* | **0.772** | **0.932** | **0.852** | **0.646** | **0.131** | **0.388** |
|  | DT | 0.956 | 0.989 | 0.973 | 0.282 | 0.057 | 0.170 |
| *C5* | ANN  ANFIS | 0.939  0.958 | 0.984  0.989 | 0.961  0.974 | 0.335  0.277 | 0.066  0.054 | 0.200  0.165 |
|  | *EvatCrop* | **0.959** | **0.989** | **0.974** | **0.274** | **0.053** | **0.163** |
|  | DT | **0.990** | **0.997** | **0.994** | **0.136** | **0.029** | **0.083** |
| *C6* | ANN  ANFIS | 0.971  0.985 | 0.993  0.996 | 0.982  0.991 | 0.231  0.165 | 0.048  0.032 | 0.139  0.099 |
|  | *EvatCrop* | 0.986 | 0.996 | 0.991 | 0.162 | 0.031 | 0.097 |
|  | DT | 0.829 | 0.952 | 0.890 | 0.559 | 0.118 | 0.338 |
| *C7* | ANN  ANFIS | 0.789  0.886 | 0.938  0.969 | 0.864  0.928 | 0.620  0.456 | 0.123  0.090 | 0.372  0.273 |
|  | *EvatCrop* | **0.888** | **0.970** | **0.929** | **0.451** | **0.089** | **0.270** |
|  | DT | **0.999** | **0.999** | **0.999** | **0.049** | **0.011** | **0.030** |
| *C8* | ANN  ANFIS | 0.965  0.990 | 0.991  0.997 | 0.978  0.994 | 0.254  0.135 | 0.057  0.027 | 0.155  0.081 |
|  | *EvatCrop* | 0.990 | 0.998 | 0.994 | 0.132 | 0.026 | 0.079 |

**RMSE* measured in mm/day.
