# Supplementary material for: EvatCrop: a novel hybrid quasi-fuzzy artificial neural network (ANN) model for estimation of reference evapotranspiration
Source: PeerJ. 2024 May 31;12:e17437. doi: 10.7717/peerj.17437 (PMC11146332; doi:10.7717/peerj.17437)
Supplement: Supplemental Information 9 [file peerj-12-17437-s009.docx]

**Table 8.** The experimental values of the performance metrics obtained for the testing set of Jayanti.

| **Input**  **combinations** | **Models** | *R*2 | *d* | *Ag* | *RMSE* | *RMSRE* | *Ae* |
| --- | --- | --- | --- | --- | --- | --- | --- |
|  | DT | 0.591 | 0.872 | 0.731 | 0.847 | 0.190 | 0.519 |
| *C*1 | ANN  ANFIS | 0.612  0.604 | 0.872  0.879 | 0.742  0.742 | 0.825  0.833 | 0.190  0.187 | 0.508  0.510 |
|  | *EvatCrop* | **0.615** | **0.880** | **0.748** | **0.821** | **0.186** | **0.504** |
|  | DT | 0.880 | 0.970 | 0.925 | 0.458 | 0.092 | 0.275 |
| *C*2 | ANN  ANFIS | 0.895  0.907 | 0.973  0.977 | 0.934  0.942 | 0.429  0.404 | 0.087  0.079 | 0.258  0.241 |
|  | *EvatCrop* | **0.909** | **0.977** | **0.943** | **0.400** | **0.078** | **0.239** |
|  | DT | 0.624 | 0.895 | 0.760 | 0.812 | 0.182 | 0.497 |
| *C*3 | ANN  ANFIS | 0.621  0.630 | 0.893  0.901 | 0.757  0.766 | 0.815  0.805 | 0.179  0.180 | 0.497  0.493 |
|  | *EvatCrop* | **0.667** | **0.907** | **0.787** | **0.764** | **0.171** | **0.467** |
|  | DT | 0.664 | 0.900 | 0.782 | 0.767 | 0.161 | 0.464 |
| *C*4 | ANN  ANFIS | 0.698  0.685 | 0.910  0.909 | 0.804  0.797 | 0.728  0.743 | 0.153  0.155 | 0.440  0.449 |
|  | *EvatCrop* | **0.704** | **0.914** | **0.809** | **0.721** | **0.150** | **0.435** |
|  | DT | 0.882 | 0.970 | 0.926 | 0.456 | 0.092 | 0.274 |
| *C*5 | ANN  ANFIS | 0.904  0.891 | 0.976  0.972 | 0.940  0.931 | 0.410  0.438 | 0.082  0.091 | 0.246  0.264 |
|  | *EvatCrop* | **0.913** | **0.978** | **0.945** | **0.390** | **0.077** | **0.234** |
|  | DT | 0.930 | 0.982 | 0.956 | 0.350 | 0.071 | 0.210 |
| *C*6 | ANN  ANFIS | 0.958  0.966 | 0.989  0.992 | 0.973  0.979 | 0.272  0.244 | 0.058  0.051 | 0.165  0.147 |
|  | *EvatCrop* | **0.970** | **0.993** | **0.981** | **0.229** | **0.045** | **0.137** |
|  | DT | 0.715 | 0.920 | 0.817 | 0.708 | 0.155 | 0.431 |
| *C*7 | ANN  ANFIS | 0.738  0.225 | 0.927  0.823 | 0.832  0.524 | 0.679  1.166 | 0.145  0.265 | 0.412  0.716 |
|  | *EvatCrop* | **0.771** | **0.940** | **0.856** | **0.633** | **0.132** | **0.383** |
|  | DT | 0.923 | 0.980 | 0.952 | 0.367 | 0.076 | 0.222 |
| *C*8 | ANN  ANFIS | 0.949  0.505 | 0.987  0.803 | 0.968  0.654 | 0.299  1.324 | 0.068  0.273 | 0.184  0.798 |
|  | *EvatCrop* | **0.972** | **0.993** | **0.982** | **0.223** | **0.045** | **0.134** |

**RMSE* measured in mm/day.
