# Supplementary material for: EvatCrop: a novel hybrid quasi-fuzzy artificial neural network (ANN) model for estimation of reference evapotranspiration
Source: PeerJ. 2024 May 31;12:e17437. doi: 10.7717/peerj.17437 (PMC11146332; doi:10.7717/peerj.17437)
Supplement: Supplemental Information 10 [file peerj-12-17437-s010.docx]

**Table 9.** The experimental values of the performance metrics obtained for the training set of Tamaguri.

| **Input**  **combinations** | **Models** | *R*2 | *d* | *Ag* | *RMSE* | *RMSRE* | *Ae* |
| --- | --- | --- | --- | --- | --- | --- | --- |
|  | DT | 0.559 | 0.842 | 0.701 | 1.043 | 0.219 | 0.631 |
| *C1* | ANN  ANFIS | 0.503  0.524 | 0.810  0.824 | 0.656  0.674 | 1.108  1.084 | 0.239  0.227 | 0.673  0.655 |
|  | *EvatCrop* | **0.560** | **0.843** | **0.701** | **1.042** | **0.218** | **0.630** |
|  | DT | 0.970 | 0.992 | 0.981 | 0.272 | 0.055 | 0.163 |
| *C2* | ANN  ANFIS | 0.957  0.968 | 0.989  0.992 | 0.973  0.980 | 0.327  0.280 | 0.071  0.057 | 0.199  0.169 |
|  | *EvatCrop* | **0.972** | **0.993** | **0.982** | **0.265** | **0.053** | **0.159** |
|  | DT | 0.642 | 0.882 | 0.762 | 0.939 | 0.189 | 0.564 |
| *C3* | ANN  ANFIS | 0.581  0.651 | 0.854  0.885 | 0.717  0.768 | 1.017  0.928 | 0.203  0.182 | 0.610  0.555 |
|  | *EvatCrop* | **0.678** | **0.897** | **0.787** | **0.891** | **0.175** | **0.533** |
|  | DT | 0.604 | 0.864 | 0.734 | 0.988 | 0.201 | 0.595 |
| *C4* | ANN  ANFIS | 0.558  0.619 | 0.843  0.871 | 0.701  0.745 | 1.044  0.970 | 0.215  0.197 | 0.629  0.583 |
|  | *EvatCrop* | **0.659** | **0.888** | **0.773** | **0.918** | **0.184** | **0.551** |
|  | DT | 0.970 | 0.992 | 0.981 | 0.271 | 0.055 | 0.163 |
| *C5* | ANN  ANFIS | 0.951  0.974 | 0.987  0.993 | 0.969  0.984 | 0.347  0.252 | 0.075  0.051 | 0.211  0.151 |
|  | *EvatCrop* | **0.976** | **0.994** | **0.985** | **0.244** | **0.049** | **0.146** |
|  | DT | 0.978 | 0.994 | 0.986 | 0.231 | 0.047 | 0.139 |
| *C6* | ANN  ANFIS | 0.976  0.988 | 0.994  0.997 | 0.985  0.993 | 0.243  0.171 | 0.048  0.034 | 0.145  0.102 |
|  | *EvatCrop* | **0.990** | **0.997** | **0.994** | **0.159** | **0.031** | **0.095** |
|  | DT | 0.676 | 0.896 | 0.786 | 0.894 | 0.181 | 0.537 |
| *C7* | ANN  ANFIS | 0.627  0.741 | 0.873  0.921 | 0.750  0.831 | 0.960  0.800 | 0.186  0.152 | 0.573  0.476 |
|  | *EvatCrop* | **0.746** | **0.923** | **0.835** | **0.791** | **0.151** | **0.471** |
|  | DT | 0.992 | 0.998 | 0.995 | 0.141 | 0.029 | 0.085 |
| *C8* | ANN  ANFIS | 0.956  0.992 | 0.988  0.998 | 0.972  0.995 | 0.331  0.143 | 0.075  0.029 | 0.203  0.086 |
|  | *EvatCrop* | **0.992** | **0.998** | **0.995** | **0.139** | **0.027** | **0.083** |

**RMSE* measured in mm/day.
