# Supplementary material for: EvatCrop: a novel hybrid quasi-fuzzy artificial neural network (ANN) model for estimation of reference evapotranspiration
Source: PeerJ. 2024 May 31;12:e17437. doi: 10.7717/peerj.17437 (PMC11146332; doi:10.7717/peerj.17437)
Supplement: Supplemental Information 11 [file peerj-12-17437-s011.docx]

**Table 10.** The experimental values of the performance metrics obtained for the testing set of Tamaguri.

| **Input**  **combinations** | **Models** | *R*2 | *d* | *Ag* | *RMSE* | *RMSRE* | *Ae* |
| --- | --- | --- | --- | --- | --- | --- | --- |
|  | DT | 0.480 | 0.802 | 0.641 | 1.099 | 0.220 | 0.659 |
| *C*1 | ANN  ANFIS | 0.501  0.499 | 0.803  0.805 | 0.652  0.652 | 1.077  1.079 | 0.214  0.216 | 0.646  0.648 |
|  | *EvatCrop* | **0.510** | **0.811** | **0.661** | **1.067** | **0.216** | **0.641** |
|  | DT | 0.939 | 0.984 | 0.961 | 0.377 | 0.073 | 0.225 |
| *C*2 | ANN  ANFIS | 0.953  0.959 | 0.988  0.989 | 0.970  0.974 | 0.331  0.307 | 0.068  0.060 | 0.199  0.183 |
|  | *EvatCrop* | **0.963** | **0.990** | **0.976** | **0.295** | **0.057** | **0.176** |
|  | DT | 0.495 | 0.818 | 0.657 | 1.083 | 0.209 | 0.646 |
| *C*3 | ANN  ANFIS | 0.541  0.541 | 0.826  0.835 | 0.684  0.688 | 1.033  1.033 | 0.197  0.199 | 0.615  0.616 |
|  | *EvatCrop* | **0.556** | **0.837** | **0.696** | **1.016** | **0.195** | **0.606** |
|  | DT | 0.531 | 0.833 | 0.682 | 1.044 | 0.208 | 0.626 |
| *C*4 | ANN  ANFIS | 0.540  0.547 | 0.832  0.842 | 0.686  0.695 | 1.034  1.026 | 0.203  0.205 | 0.619  0.615 |
|  | *EvatCrop* | **0.569** | **0.847** | **0.708** | **1.000** | **0.201** | **0.601** |
|  | DT | 0.935 | 0.983 | 0.959 | 0.388 | 0.075 | 0.231 |
| *C*5 | ANN  ANFIS | 0.948  0.953 | 0.987  0.988 | 0.967  0.971 | 0.349  0.330 | 0.068  0.068 | 0.208  0.199 |
|  | *EvatCrop* | **0.965** | **0.991** | **0.978** | **0.284** | **0.055** | **0.170** |
|  | DT | 0.955 | 0.989 | 0.972 | 0.322 | 0.062 | 0.192 |
| *C*6 | ANN  ANFIS | 0.975  0.972 | 0.994  0.993 | 0.984  0.982 | 0.240  0.257 | 0.047  0.055 | 0.143  0.156 |
|  | *EvatCrop* | **0.985** | **0.996** | **0.990** | **0.190** | **0.037** | **0.113** |
|  | DT | 0.549 | 0.845 | 0.697 | 1.024 | 0.201 | 0.613 |
| *C*7 | ANN  ANFIS | 0.586  0.305 | 0.852  0.792 | 0.719  0.548 | 0.981  1.271 | 0.182  0.275 | 0.582  0.773 |
|  | *EvatCrop* | **0.620** | **0.871** | **0.745** | **0.940** | **0.175** | **0.558** |
|  | DT | 0.956 | 0.988 | 0.973 | 0.318 | 0.061 | 0.190 |
| *C*8 | ANN  ANFIS | 0.954  0.916 | 0.988  0.979 | 0.971  0.948 | 0.328  0.441 | 0.071  0.111 | 0.200  0.276 |
|  | *EvatCrop* | **0.986** | **0.996** | **0.991** | **0.183** | **0.036** | **0.110** |

**RMSE* measured in mm/day.
