# Supplementary figures and images for: EvatCrop: a novel hybrid quasi-fuzzy artificial neural network (ANN) model for estimation of reference evapotranspiration
Source: PeerJ. 2024 May 31;12:e17437. doi: 10.7717/peerj.17437 (PMC11146332; doi:10.7717/peerj.17437)

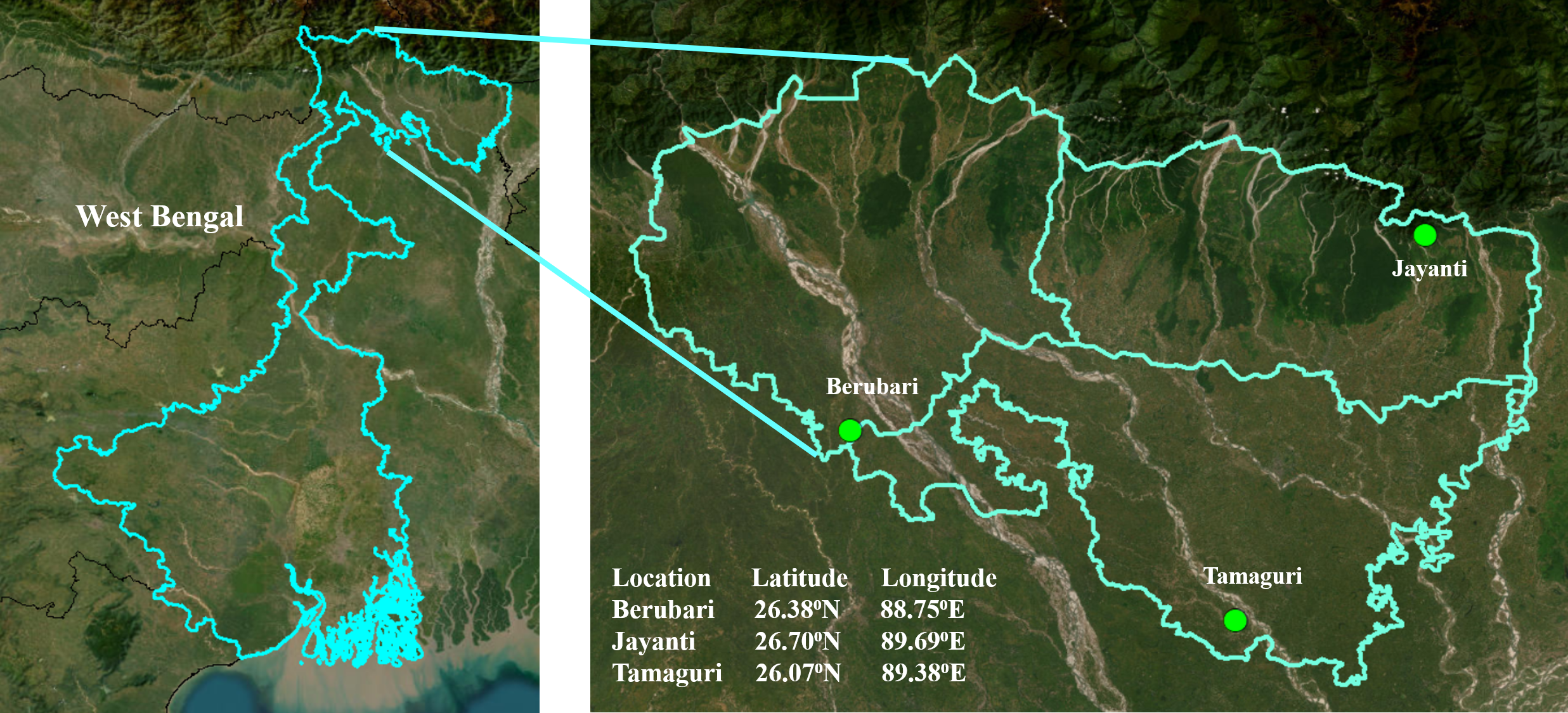

Supplement: Supplemental Information 12 — ESRI (2009). Imagery [basemap], Scale Not Given. World Imagery. 13 Dec, 2009 (Url:https://www.arcgis.com/home/item.html?id=10df2279f9684e4a9f6a7f08febac2a9) [file peerj-12-17437-s012.png]

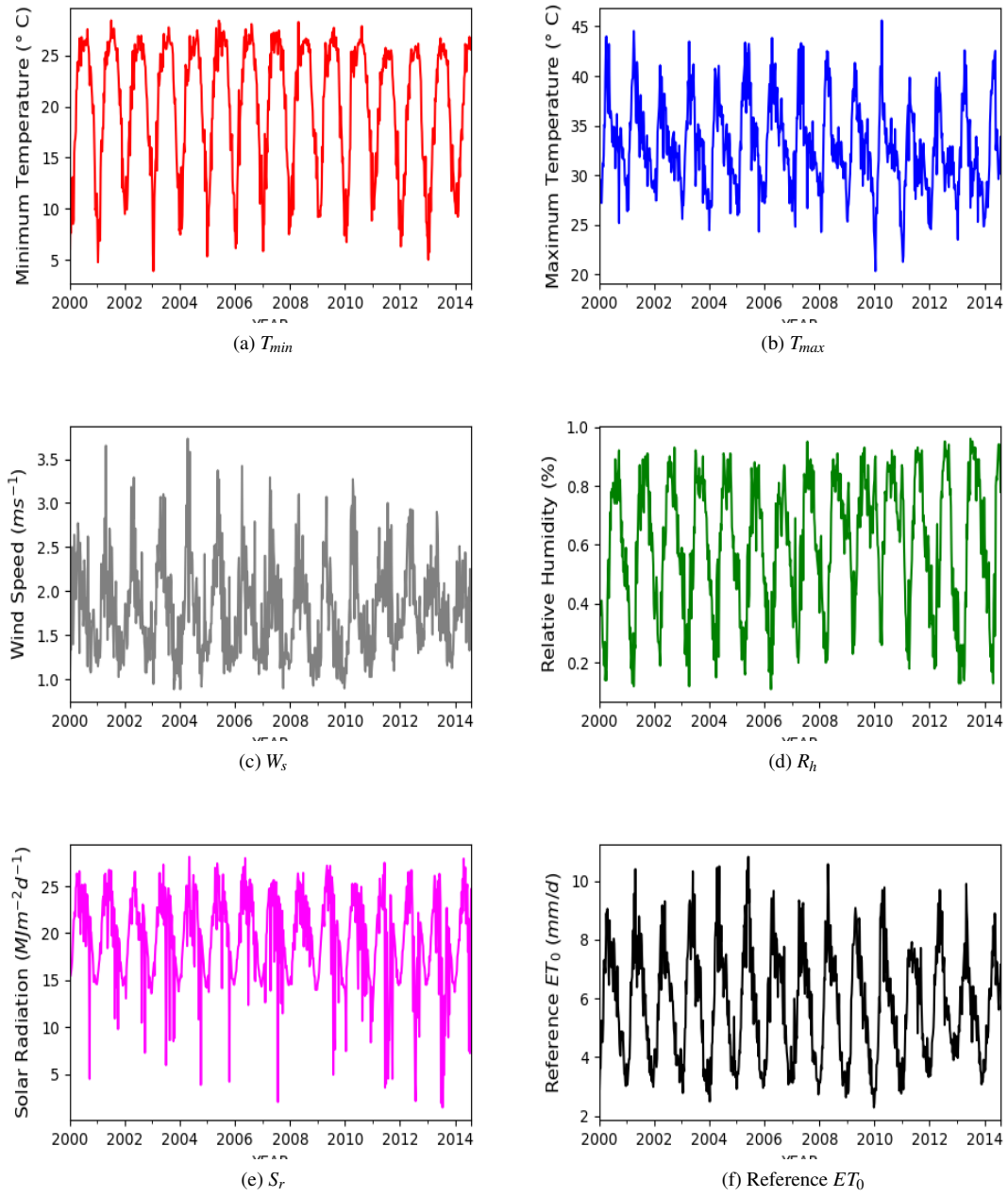

Figure 1: Time series plots for each of the input parameters and reference  $ET_0$  for Berubari

Supplement: Supplemental Information 13 [file peerj-12-17437-s013.pdf]

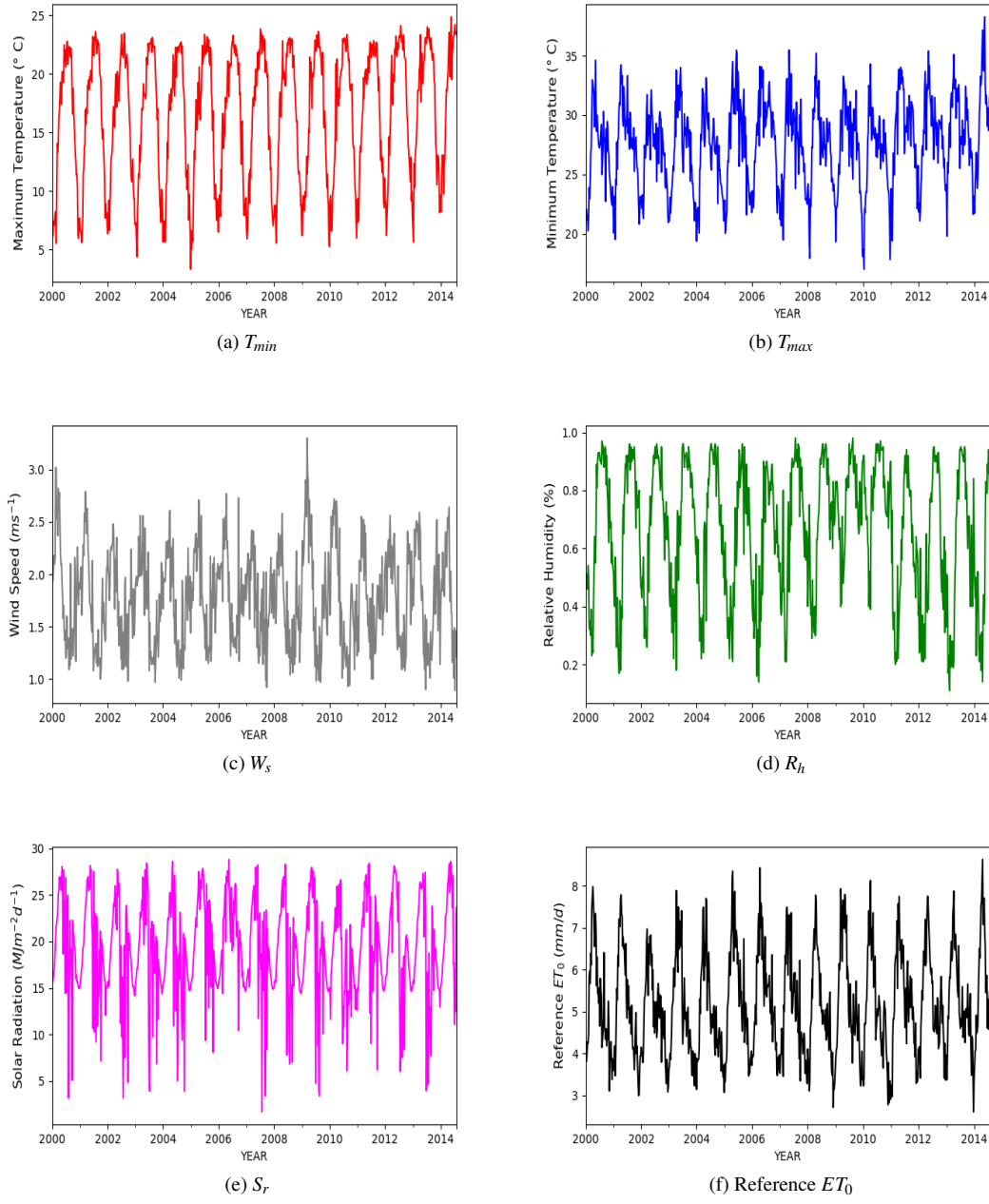

Figure 2: Time series plots for each of the input parameters and reference  $ET_0$  for Jayanti

Supplement: Supplemental Information 14 [file peerj-12-17437-s014.pdf]

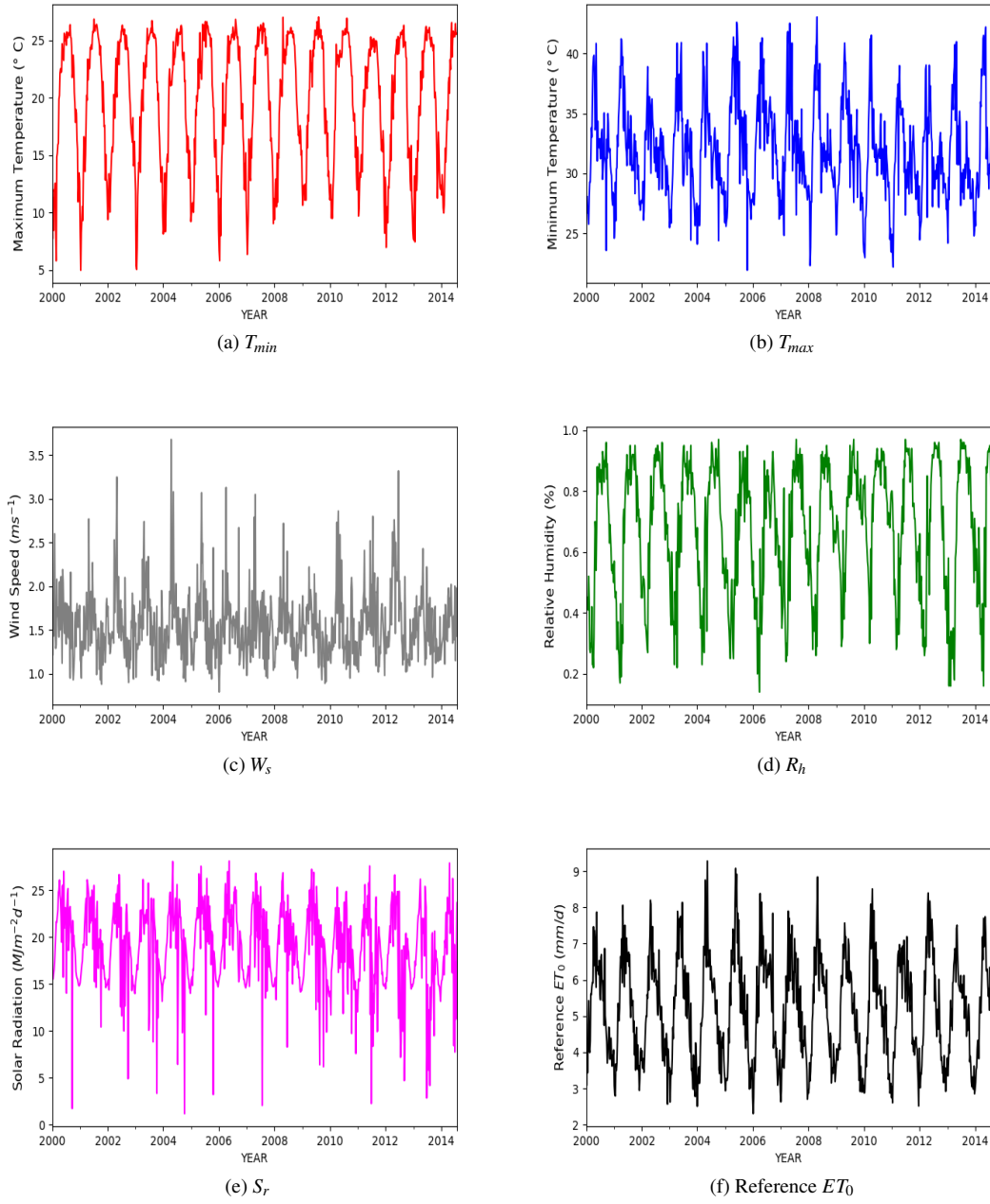

Figure 3: Time series plots for each of the input parameters and reference  $ET_0$  for Tamaguri

Supplement: Supplemental Information 15 [file peerj-12-17437-s015.pdf]

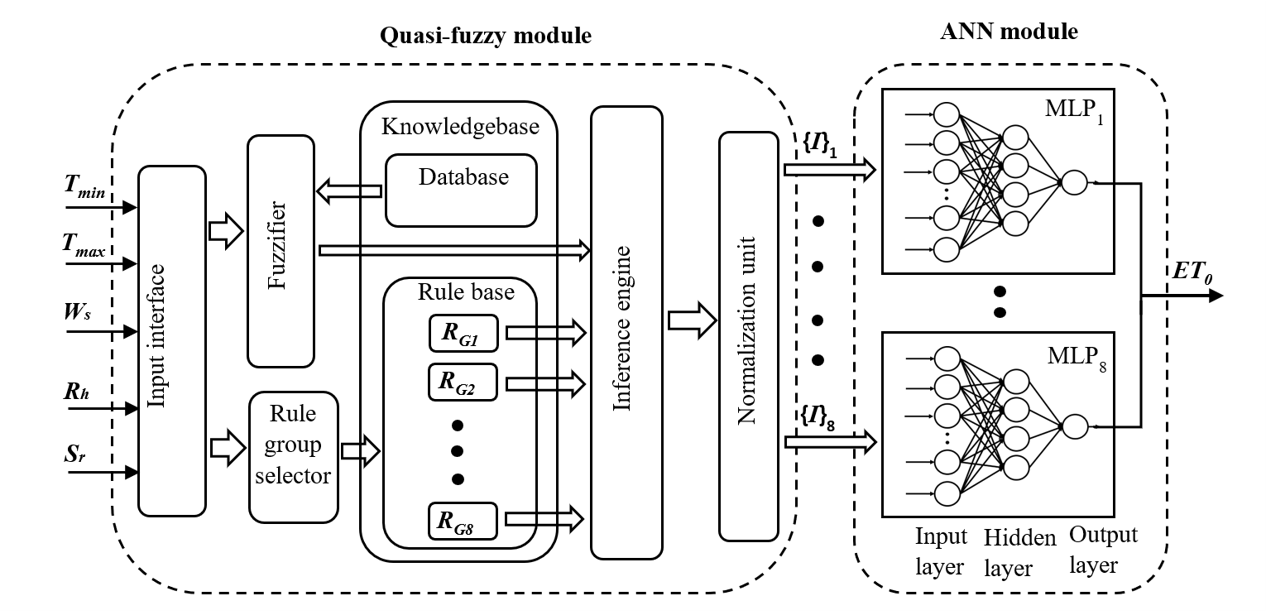

Supplement: Supplemental Information 16 [file peerj-12-17437-s016.png]

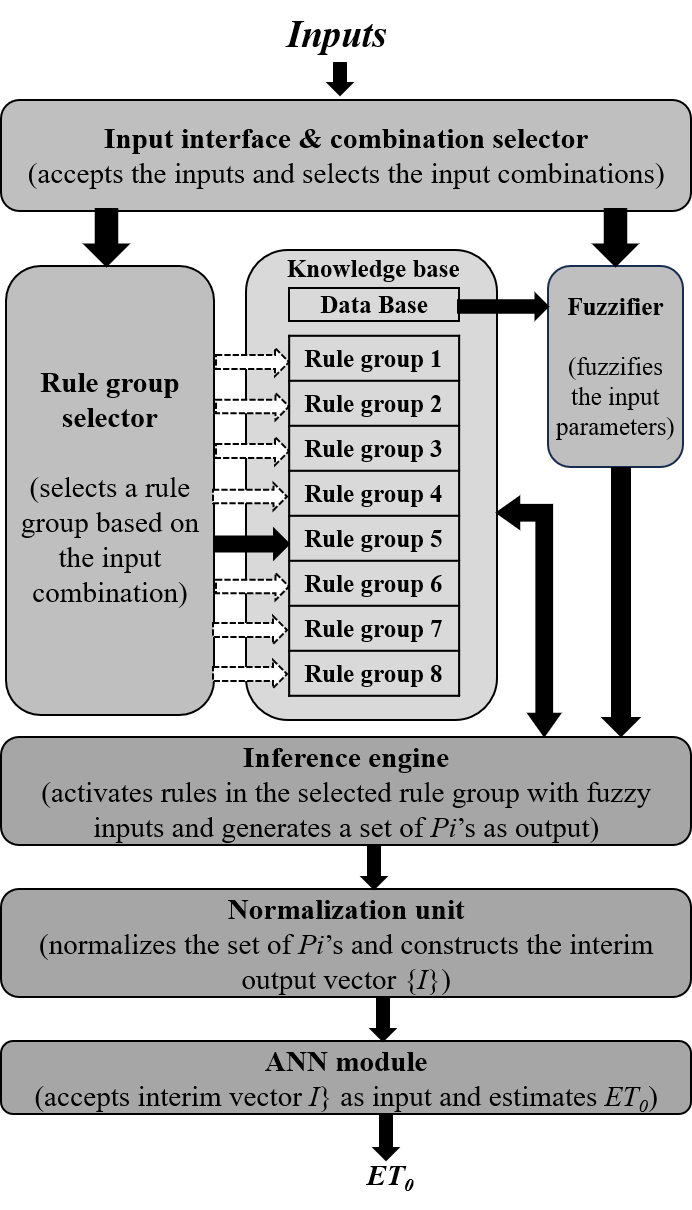

Supplement: Supplemental Information 17 [file peerj-12-17437-s017.png]
