# Supplementary material for: EvatCrop: a novel hybrid quasi-fuzzy artificial neural network (ANN) model for estimation of reference evapotranspiration
Source: PeerJ. 2024 May 31;12:e17437. doi: 10.7717/peerj.17437 (PMC11146332; doi:10.7717/peerj.17437)
Supplement: Supplemental Information 18 [file peerj-12-17437-s018.pdf]

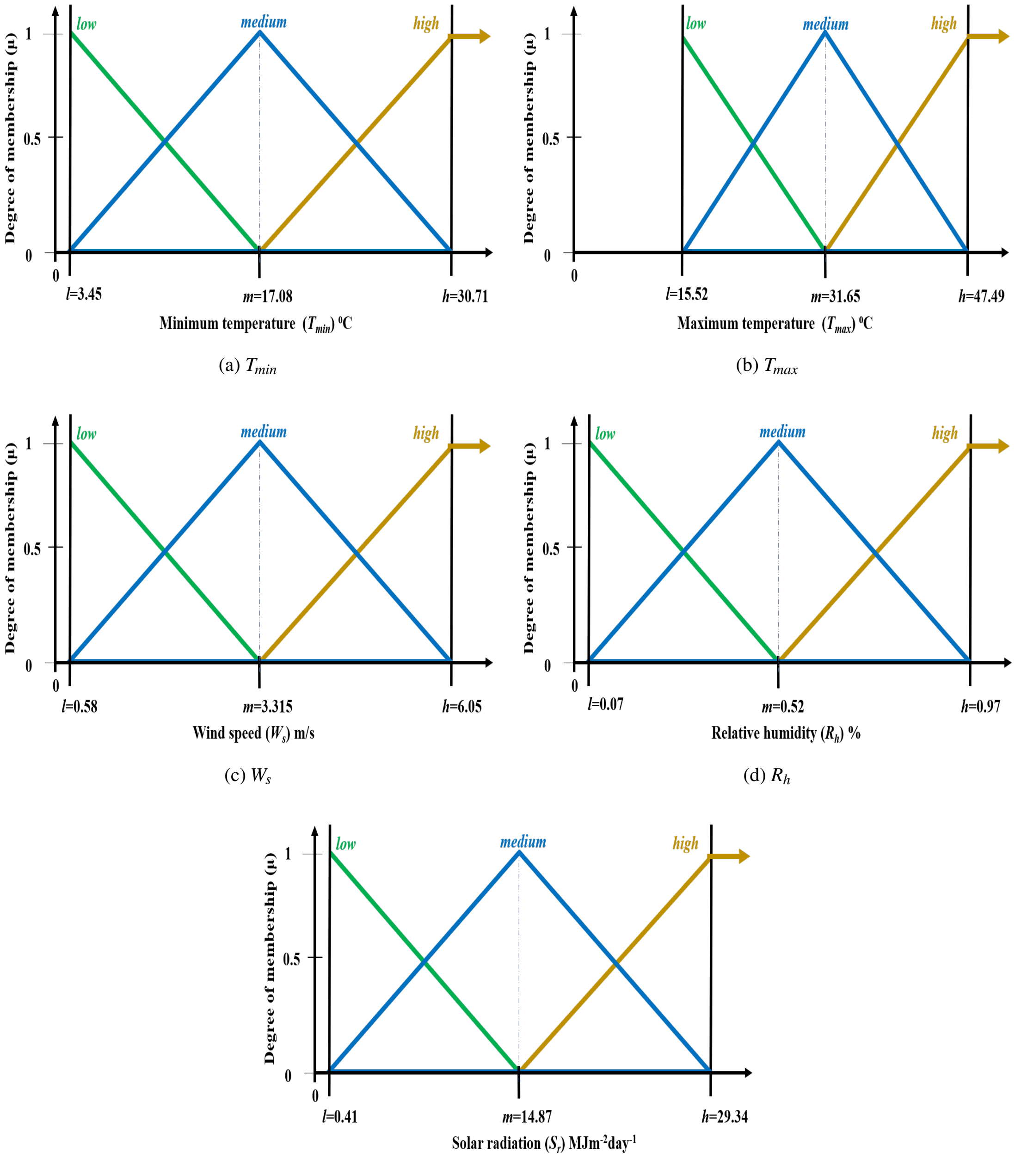

(a) T_{min}

(b) T_{max}

(c) W_s

(d) R_h

(e) S_r

Figure 7: The shapes and range of values of fuzzy membership functions of each input parameter for Berubari
