# Supplementary material for: EvatCrop: a novel hybrid quasi-fuzzy artificial neural network (ANN) model for estimation of reference evapotranspiration
Source: PeerJ. 2024 May 31;12:e17437. doi: 10.7717/peerj.17437 (PMC11146332; doi:10.7717/peerj.17437)
Supplement: Supplemental Information 21 [file peerj-12-17437-s021.pdf]

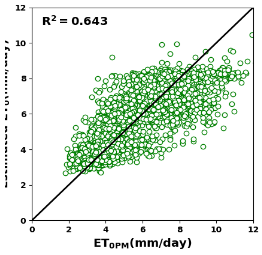

(a) $C1$ (Training set)

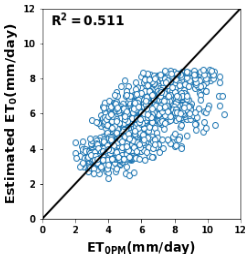

(b) $C1$ (Testing set)

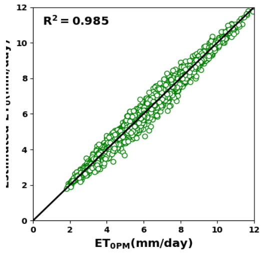

(c) $C2$ (Training set)

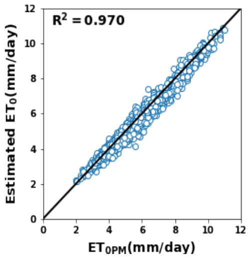

(d) $C2$ (Testing set)

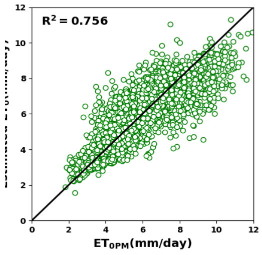

(e) $C3$ (Training set)

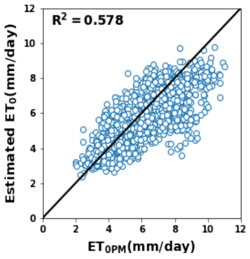

(f) $C3$ (Testing set)

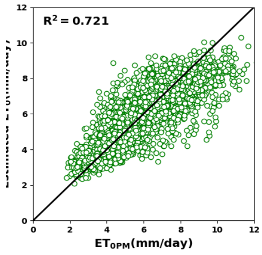

(g) $C4$ (Training set)

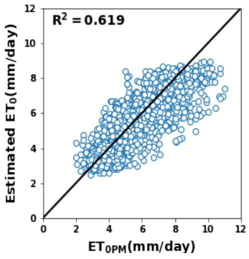

(h) $C4$ (Testing set)

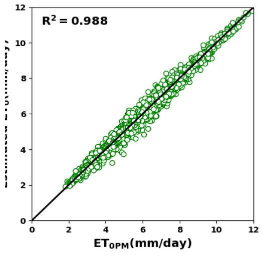

(i) $C5$ (Training set)

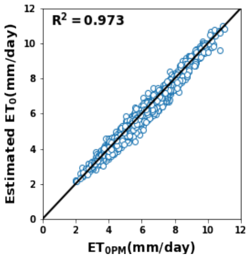

(j) $C5$ (Testing set)

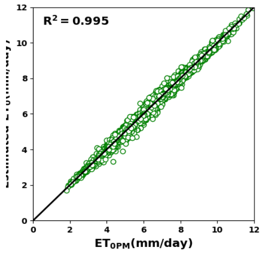

(k) $C6$ (Training set)

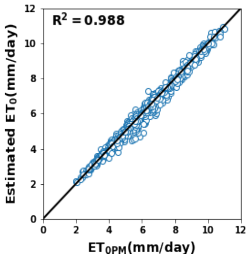

(l) $C6$ (Testing set)

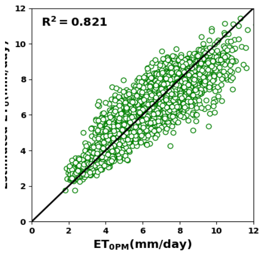

(m) $C7$ (Training set)

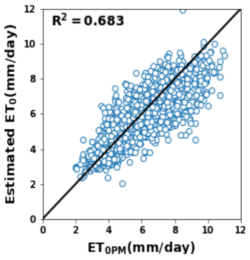

(n) $C7$ (Testing set)

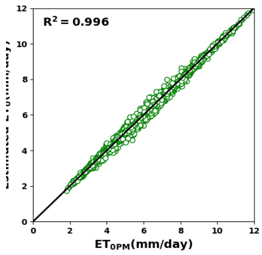

(o) $C8$ (Testing set)

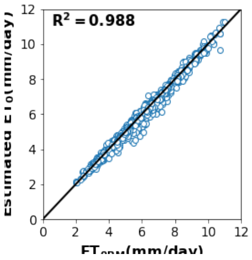

(p) $C8$ (Testing set)

Figure 10: Scatter plots of estimated ET_0 against ET_{0PM} obtained for the training and testing sets of Berubari
