# Supplementary material for: EvatCrop: a novel hybrid quasi-fuzzy artificial neural network (ANN) model for estimation of reference evapotranspiration
Source: PeerJ. 2024 May 31;12:e17437. doi: 10.7717/peerj.17437 (PMC11146332; doi:10.7717/peerj.17437)
Supplement: Supplemental Information 26 [file peerj-12-17437-s026.pdf]

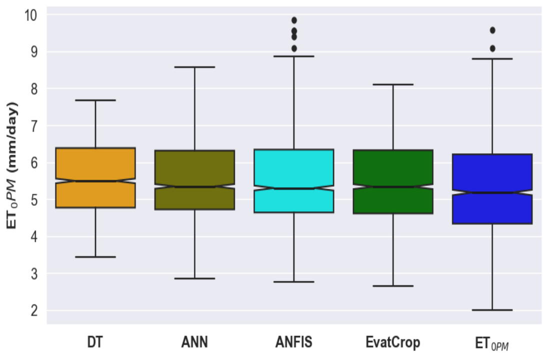

(a) *C1*

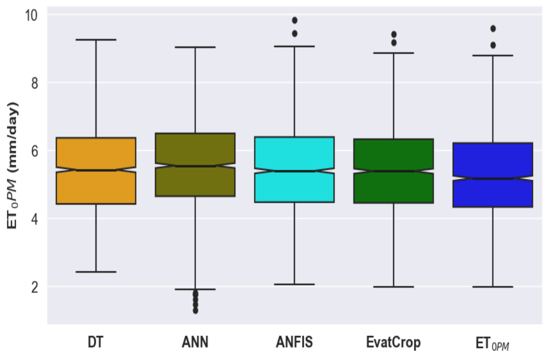

(b) *C2*

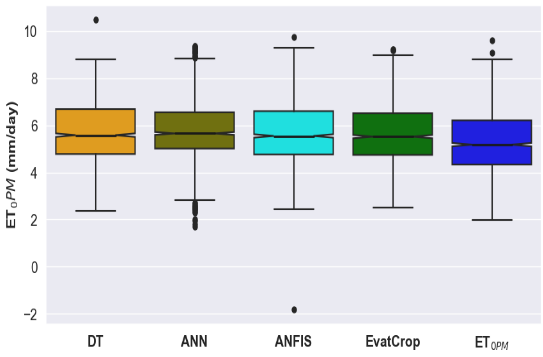

(c) *C3*

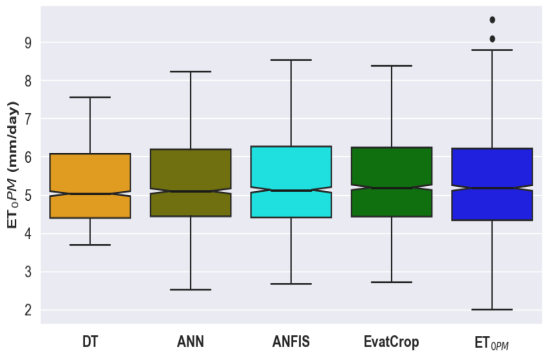

(d) *C4*

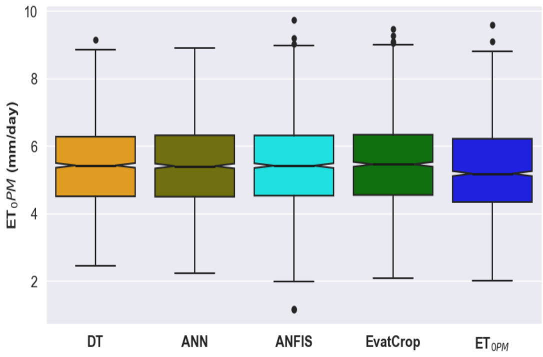

(e) *C5*

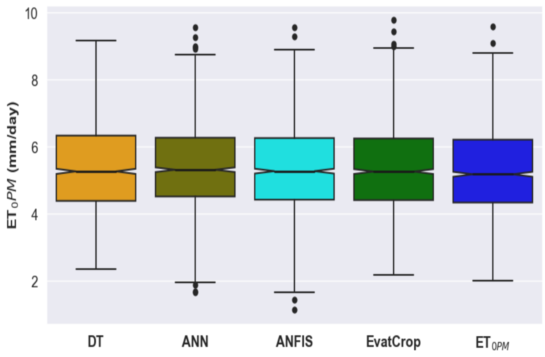

(f) *C6*

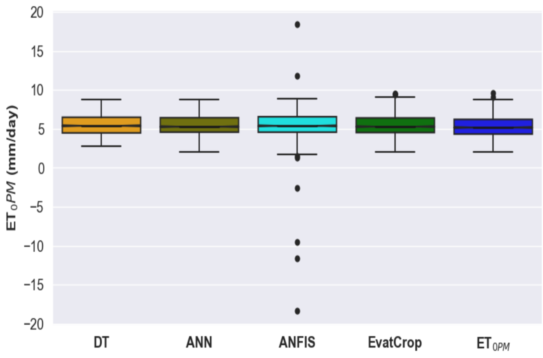

(g) *C7*

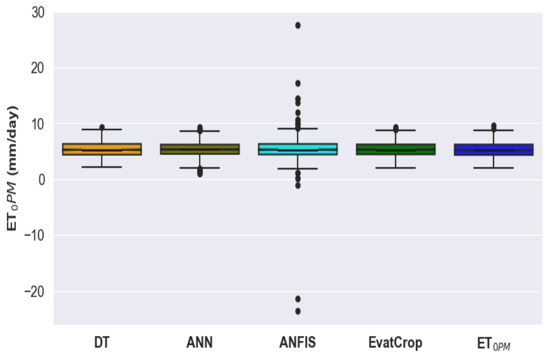

(h) *C8*

Figure 15: Boxplots for estimated ET_0 of DT, ANN, ANFIS, *EvatCrop*, and ET_{0PM} obtained for the testing set of Jayanti
