# Supplementary material for: EvatCrop: a novel hybrid quasi-fuzzy artificial neural network (ANN) model for estimation of reference evapotranspiration
Source: PeerJ. 2024 May 31;12:e17437. doi: 10.7717/peerj.17437 (PMC11146332; doi:10.7717/peerj.17437)
Supplement: Supplemental Information 27 [file peerj-12-17437-s027.pdf]

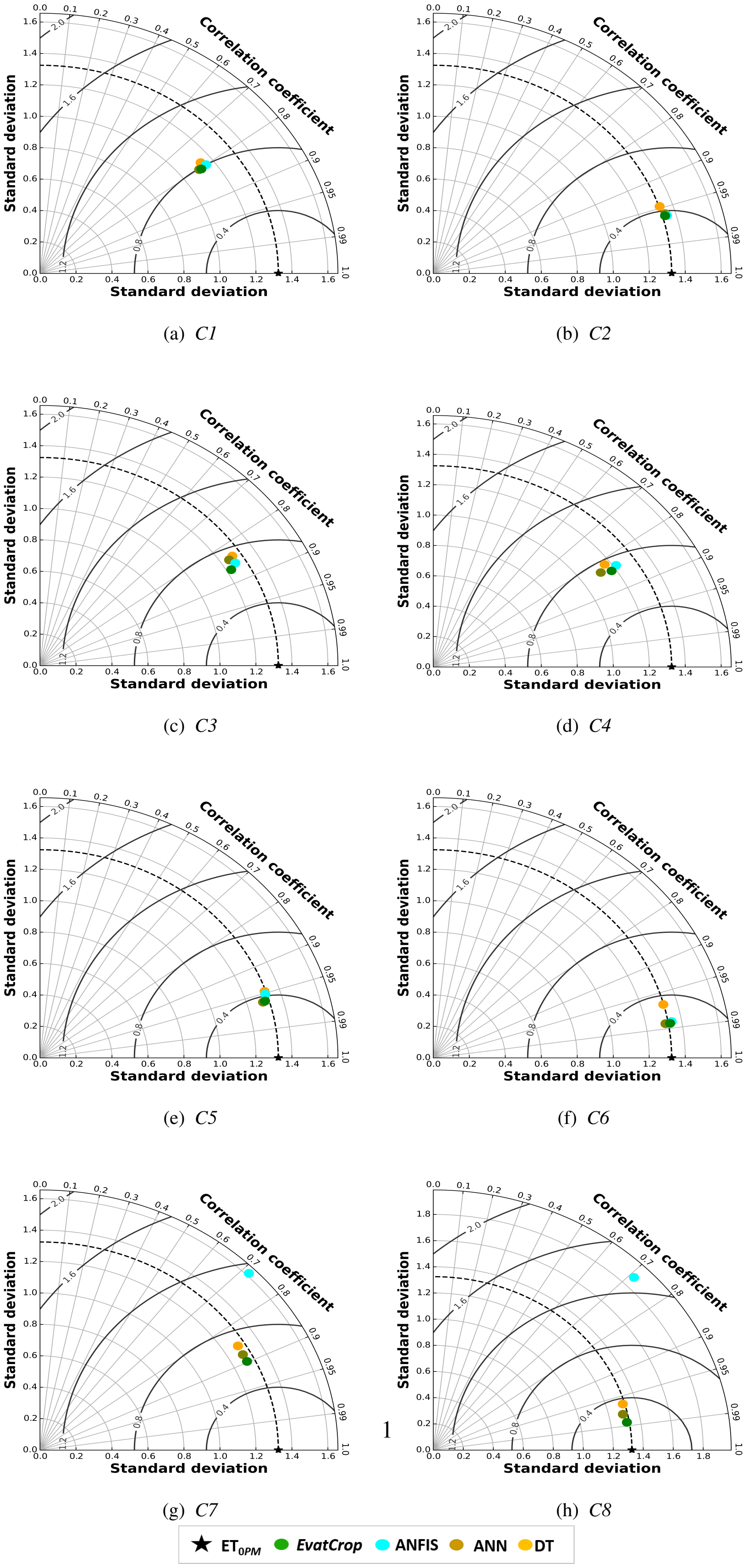

(a) *C1* (b) *C2*

(c) *C3* (d) *C4*

(e) *C5* (f) *C6*

(g) *C7* (h) *C8*

Figure 16: Taylor diagrams for estimated ET_0 of DT, ANN, ANFIS, *EvatCrop*, and ET_{0PM} obtained for the testing set of Jayanti
