# Supplementary material for: EvatCrop: a novel hybrid quasi-fuzzy artificial neural network (ANN) model for estimation of reference evapotranspiration
Source: PeerJ. 2024 May 31;12:e17437. doi: 10.7717/peerj.17437 (PMC11146332; doi:10.7717/peerj.17437)
Supplement: Supplemental Information 29 [file peerj-12-17437-s029.pdf]

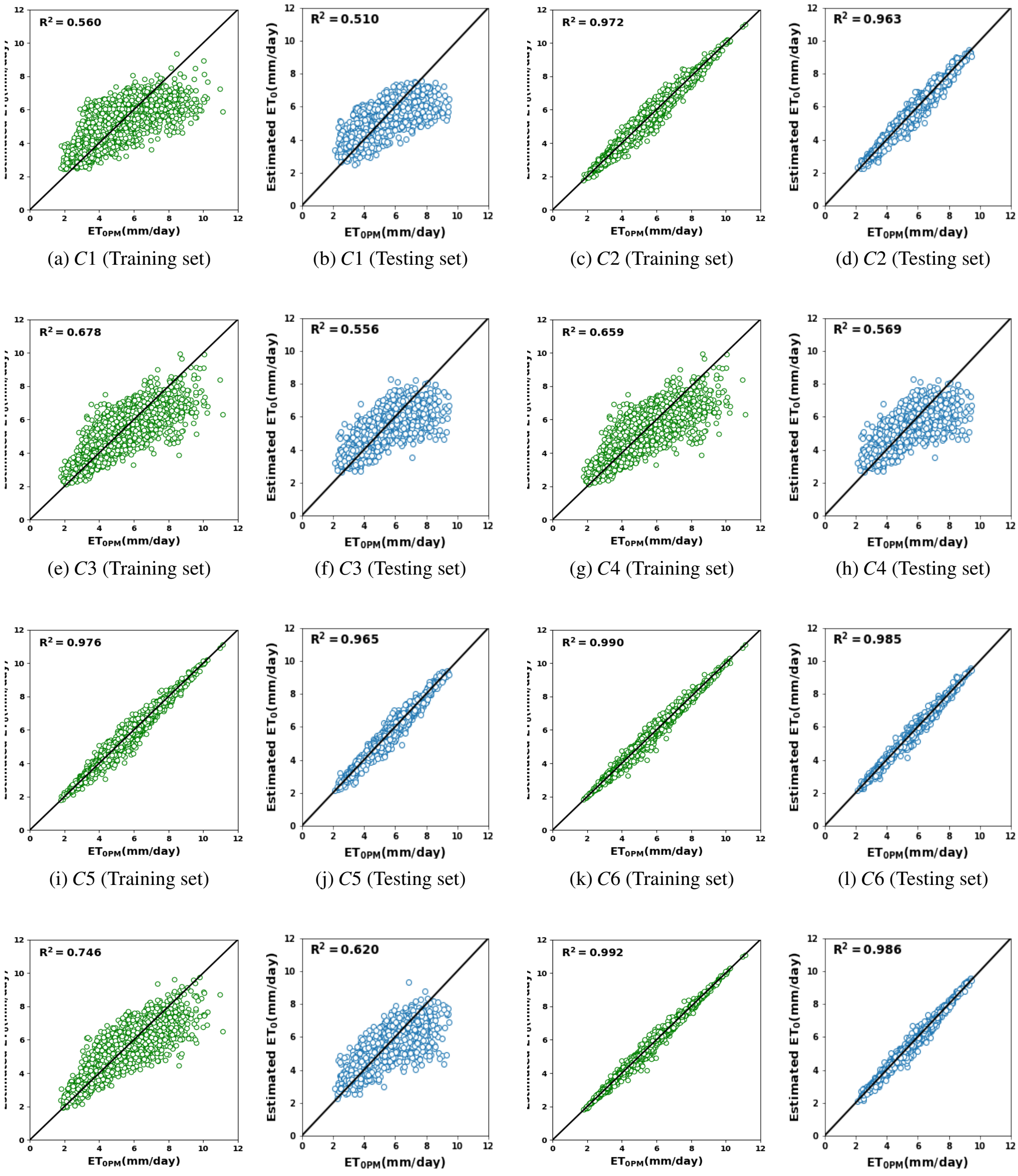

(a) $C1$ (Training set) (b) $C1$ (Testing set) (c) $C2$ (Training set) (d) $C2$ (Testing set)

(e) $C3$ (Training set) (f) $C3$ (Testing set) (g) $C4$ (Training set) (h) $C4$ (Testing set)

(i) $C5$ (Training set) (j) $C5$ (Testing set) (k) $C6$ (Training set) (l) $C6$ (Testing set)

(m) $C7$ (Training set) (n) $C7$ (Testing set) (o) $C8$ (Testing set) (p) $C8$ (Testing set)

Figure 18: Scatter plots of estimated ET_0 against ET_{0PM} obtained for the training and testing sets of Tamaguri
