# Supplementary material for: EvatCrop: a novel hybrid quasi-fuzzy artificial neural network (ANN) model for estimation of reference evapotranspiration
Source: PeerJ. 2024 May 31;12:e17437. doi: 10.7717/peerj.17437 (PMC11146332; doi:10.7717/peerj.17437)
Supplement: Supplemental Information 32 [file peerj-12-17437-s032.pdf]

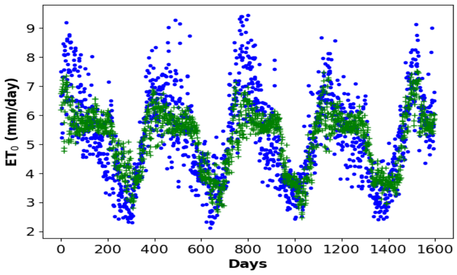


(a) *C1*

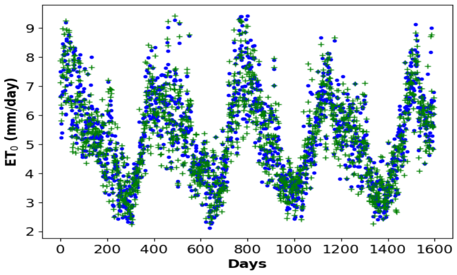


(b) *C2*

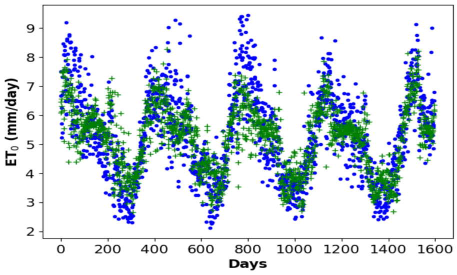


(c) *C3*

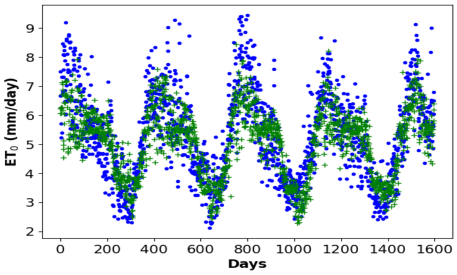


(d) *C4*

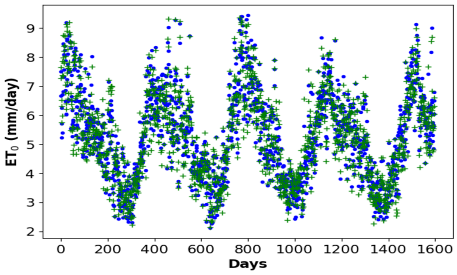


(e) *C5*

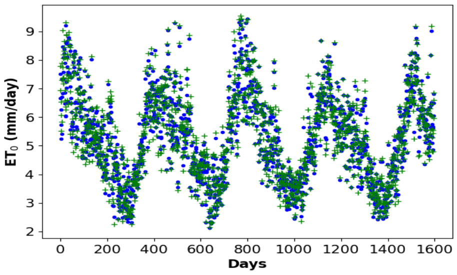


(f) *C6*

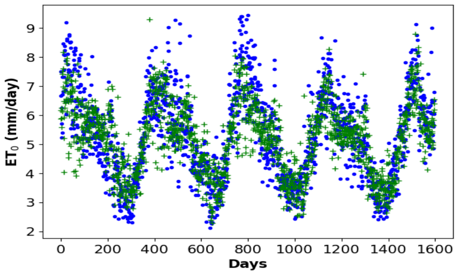


(g) *C7*

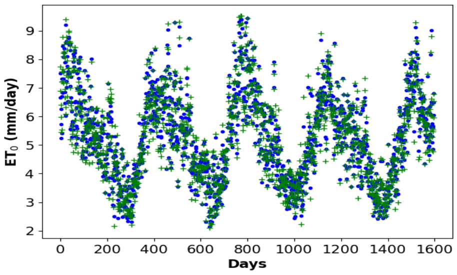


(h) *C8*

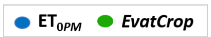


Figure 21: Distribution plots for estimated $ET_0$ of *EvatCrop*, and $ET_{0PM}$ obtained for the testing set of Tamaguri
